# Interplay between Nox2 Activity and Platelet Activation in Patients with Sepsis and Septic Shock: A Prospective Study

**DOI:** 10.1155/2020/4165358

**Published:** 2020-10-27

**Authors:** Giusy Tiseo, Elena Cavarretta, Arianna Forniti, Cristina Nocella, Sebastiano Sciarretta, Ornella Spagnolello, Enrico Baldini, Mariangela Peruzzi, Giuliano Bertazzoni, Francesco Menichetti, Antonino G. M. Marullo, Fabio Miraldi, Andrea Morelli, Giacomo Frati, Roberto Carnevale, Marco Falcone

**Affiliations:** ^1^Department of Clinical and Experimental Medicine, University of Pisa, Pisa, Italy; ^2^Mediterranea Cardiocentro, Napoli, Italy; ^3^Department of Medico-Surgical Sciences and Biotechnologies, Sapienza University of Rome, Latina, Italy; ^4^Department of Internal Clinical, Anesthesiological and Cardiovascular Sciences, Sapienza University of Rome, Rome, Italy; ^5^Department of Angio-Cardio-Neurology, IRCCS NeuroMed, Pozzilli, Italy; ^6^Emergency Department, Policlinico Umberto I Hospital, Sapienza University, Rome, Italy

## Abstract

**Background:**

Although preclinical studies highlighted the potential role of NADPH oxidase (NOX) in sepsis, only few studies evaluated the oxidative stress in patients with sepsis and septic shock. The objective of the study is to appraise the oxidative stress status and platelet function in patients with sepsis and septic shock compared to healthy controls.

**Methods and Results:**

Patients with sepsis or septic shock admitted to the hospital Policlinico Umberto I (Sapienza University, Rome) underwent a blood sample collection within 1 hour from admission. Platelet aggregation, serum thromboxane B2 (TxB2), soluble NOX2-derived peptides (sNox2-dp), and hydrogen peroxide breakdown activity (HBA) were measured and compared to those of healthy volunteers. Overall, 33 patients were enrolled; of these, 20 (60.6%) had sepsis and 13 (39.4%) septic shock. Compared to healthy controls (*n* = 10, age 67.8 ± 3.2, male 50%), patients with sepsis and septic shock had higher platelet aggregation (49% (IQR 45-55), 60% (55.75-67.25), and 73% (IQR 69-80), respectively, *p* < 0.001), higher serum TxB2 (77.5 (56.5-86.25), 122.5 (114-131.5), and 210 (195-230) pmol/L, respectively, *p* < 0.001), higher sNox2-dp (10 (7.75-12), 19.5 (17.25-21), and 33 (29.5-39) pg/mL, respectively, *p* < 0.001), and lower HBA (75% (67.25-81.5), 50% (45-54.75), and 27% (21.5-32.5), respectively, *p* < 0.001). Although not statistically significant, a trend in higher levels of serum TxB2 and sNox2-dp in patients who died was observed.

**Conclusions:**

Patients with septic shock exhibit higher Nox2 activity and platelet activation than patients with sepsis. These insights joined to better knowledge of these mechanisms could guide the identification of future prognostic biomarkers and new therapeutic strategies in the scenario of septic shock.

## 1. Introduction

Sepsis is an increasing cause of admission to the Emergency Department (ED) and is associated with mortality rates potentially exceeding 50% [1–4]. Pathophysiological derangements occurring during sepsis, such as endothelial dysfunction, increased nitric oxide and arachidonic acid derivative synthesis (such as thromboxane and isoprostanes), and activation of inflammatory patterns, are responsible for the dysregulated host response and development of organ damage [5]. Platelets play a strategic role in the elicitation of the immune response to sepsis, being equipped with specific receptors able to respond to pathogens and enzyme systems capable of producing reactive oxygen species (ROS), such as NADPH oxidase (NOX) [6]. The interplay between the oxidative stress and platelet activation [7–9] may contribute to the alterations occurring during sepsis and septic shock, and its appraisal may be useful for both identification of prognostic markers and future exploration of new therapeutic approaches. Although some preclinical evidences highlighted the potential role of the NOX signaling pathway in animal models of sepsis [10, 11], few studies evaluated the oxidative stress levels and platelet function in patients with sepsis and septic shock.

The aim of our study is to evaluate the oxidative stress status in patients with sepsis and in those with septic shock compared to healthy controls.

## 2. Methods

### 2.1. Study Design

This is a prospective study including nonconsecutive patients with sepsis and septic shock admitted to the ED of the tertiary care hospital Policlinico Umberto I, Sapienza University (Rome, Italy), from April 2017 to April 2018. The study has been performed in accordance with the ethical principles of the Declaration of Helsinki. The institutional review board at the Sapienza University of Rome approved the study protocol (Prot no. 560/17, ref 4550).

The primary goal was to evaluate the oxidative stress status and platelet function in patients with sepsis and in those with septic shock compared to healthy controls.

The secondary objective of the study was

- to compare the oxidative stress status and platelet function between septic patients who died and who survived within 30 days from the sepsis onset

In addition, we tried to identify differences in echocardiographic patterns in patients with sepsis and in those with septic shock.

Patients aged >18 years admitted to ED with sepsis or septic shock during the study period were eligible for inclusion in the study. Exclusion criteria were as follows: previous documented myocardial infarction (within 30 days), history of documented heart failure (according to ESC guidelines) [12], other causes of shock (hypovolemic, obstructive, and cardiogenic), severe heart valve disease, preexisting severe renal failure, pregnancy, active major bleeding, or Hb < 7g/L or platelets < 20 × 10^3^/mm^3^. Patients were finally included in the study in the study if the collection of a blood sample was possible within 1 hour from ED admission and before fluid replacement therapy and inotrope administration. When possible, patients underwent transthoracic echocardiography (TTE) during the first 12 h following the diagnosis of sepsis, as previously described [13]. All included patients underwent medical history record, physical examination, and blood sample collection within 1 hour from the diagnosis of sepsis/septic shock. Sepsis and septic shock have been defined according to the current definitions [14]. Demographic data, underlying diseases, and severity of illness of patients were reported on a standardized report form. Clinical data were assessed and recorded at the onset of signs of sepsis or septic shock. Data about the use of inotropes, the source of infection, and the administered antimicrobial therapy has been also recorded [15]. Study investigators prospectively followed all included patients with bedside visits every day. All patients were followed up for 30 days from the episode of sepsis or until death.

### 2.2. Laboratory Methods

Blood samples were obtained from all patients (within 1 hour from the diagnosis of sepsis/septic shock) and from 10 healthy volunteers and stored at -80°C until use with the antioxidant butylated hydroxytoluene (BHT) at a final concentration of 20 mmol/L. To obtain platelet-rich plasma (PRP), citrated blood samples (9 parts of blood and 1 part of 3.8% Na citrate) were centrifuged for 15 minutes at 180 g. To avoid leukocyte contamination, only the top 75% of the PRP was collected. In each PRP sample, platelet count was adjusted to 2 × 10^8^ platelets/mL. Platelet aggregation was evaluated on a Bio/Data 8-channel platelet aggregometer (PAP-8E, Bio/Data) using siliconized glass cuvettes under continuous stirring at 1200 rpm. Serum thromboxane B2 (TxB2) was measured by ELISA and expressed as picomoles per liter (pmol/L). Intra-assay and interassay coefficients of variation were 4.0% and 3.6%, respectively. Serum Nox2 activity (sNox2-dp) was measured by ELISA as previously described [16]. Finally, serum H_2_O_2_ breakdown activity (HBA) was measured with an HBA assay kit (Aurogene, code HPSA-50) as previously reported [17]. The % of HBA was calculated according to the following formula: %ofHBA = [(Ac − As)/Ac] × 100, where Ac is the absorbance of H_2_O_2_ 1.4 mg/mL and As is the absorbance in the presence of the serum sample.

### 2.3. Statistical Analysis

According to the aim of the study, a comparison between parameters of oxidative stress and platelet function among patients with sepsis, patients with septic shock, and healthy controls was performed. Moreover, we performed a comparison of the same variables between patients who survived and those who died after the episode of sepsis and septic shock.

Continuous variables were compared by Student's *t*-test if normally distributed and the Mann–Whitney *U* test if nonnormally distributed. Categorical variables were evaluated using *χ*^2^ or the two-tailed Fisher's exact test. Analysis of variance (ANOVA) was used to analyze the differences among groups. Multivariate analysis was performed to identify factors independently associated with 30-day mortality. Variables significant in the univariate analysis and those deemed to have clinical significance were included in the multivariate model. Platelet aggregation, serum TxB2, sNox2-dp, and serum HBA were also included to test their potential association with death. A stepwise multiple logistic regression model was finally used to identify predictors of mortality. Odds ratio (OR) and 95% confidence intervals (CI) were calculated.

Statistical significance was established at ≤0.05. All reported *p* values are 2-tailed. The results obtained were analyzed using commercially available statistical software packages (SPSS, version 20.0; SPSS, Inc., Chicago, IL, and R, version 3.0.2; R Development Core Team, Vienna, Austria).

## 3. Results

A total of 33 patients were included in the study. Thirteen (39.4%) patients had septic shock. Overall, 9 (27.3%) patients had pneumonia, 8 (24.3%) a genitourinary tract infection, 8 (24.3%) an intra-abdominal infection, 1 (3%) a skin and soft tissue infection, 1 (3%) osteomyelitis, 1 (3%) an intravascular device-related infection, and 1 (3%) meningitis. Two (6.1%) patients had a bloodstream infection from an unknown source of infection. In 23 cases, the etiological pathogen was detected. Supplementary Table 1 summarizes the sources of infection with relative etiological diagnosis.

No differences in age, comorbidities, and source of infection have been found between the two groups (Table 1). A trend to higher mortality rates was observed in patients with septic shock (46.2%) compared to those with sepsis (15%, *p* = 0.05).

**Figure 1:**
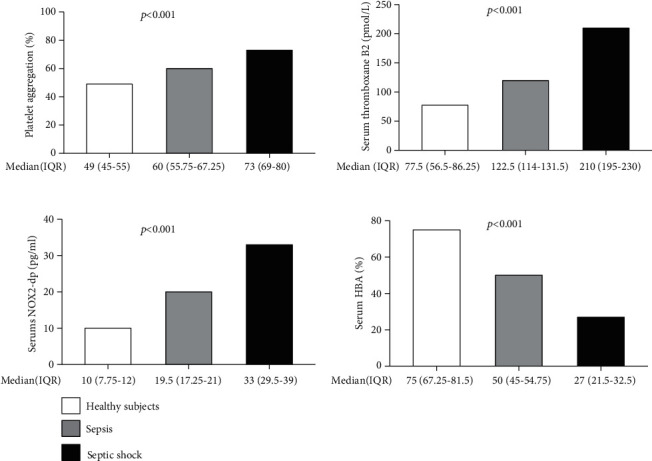
Comparison of platelet aggregation percentage, serum thromboxane B2, Nox2 activity, and serum HBA among healthy controls, patients with sepsis, and those with septic shock. Legend: ^∗^*p* < 0.001 among the 3 groups (one-way ANOVA for *k* samples). HBA = hydrogen peroxide breakdown activity; IQR = interquartile ranges; TBx2 = thromboxane B2.

Platelet aggregation was higher in patients with septic shock (73%) compared to those with sepsis (60%) and healthy controls (49%, *p* < 0.001). A similar trend has been observed for TxB2 (septic shock 210 pmol/L, sepsis 122.5 pmol/L, and healthy controls 77.5 pmol/L, *p* < 0.001) and serum Nox2-dp (septic shock 33 pg/mL, sepsis 19.5 pg/mL, and healthy controls 10 pg/mL, *p* < 0.001). Conversely, antioxidant status, evaluated by hydrogen peroxide breakdown activity (HBA), was 75% in healthy controls and lower in patients with sepsis (50%) and in those with septic shock (27%, *p* < 0.001) (Figure 1). Although not statistically significant, platelet aggregation, serum TxB2, and sNox2-dp tend to be higher in patients who died, while serum HBA levels were higher in survivors (Table 2). Multivariate analysis identified the SOFA score on admission (OR 1.34, 95% CI 1.03-1.88, *p* = 0.03) and Charlson Comorbidity Index (OR 1.48, 95% CI 1.05-2.06, *p* = 0.02) as independent predictors of 30-day mortality.

TTE was performed in 27 patients (17 with sepsis and 10 with septic shock). No significant differences were found in echocardiographic parameters, except for the inferior vena cava (IVC) collapsibility index, which was higher in patients with sepsis compared to those with septic shock (*p* = 0.035) (Supplementary Table 2). Similarly, no significant differences were found among patients who survived and did not probably due to a small sample effect (Supplementary Table 3). The only significantly different parameters were the right ventricular diameter (33 mm (30.8, 35.0) vs. 38 mm (36.5, 41.0), *p* = 0.033) and *E*/*E*′ ratio (5.8 (5.6, 6.8) vs. 7.8 (7.2, 13.2), *p* = 0.037) being both higher in patients who died.

## 4. Discussion

Patients with septic shock exhibit higher Nox2 activity and impaired systemic scavenging capacity of H_2_O_2_ when compared to patients with sepsis and to healthy controls. Moreover, they have higher serum TxB2, a reliable marker of platelet aggregation.

The increasing upregulation of these parameters from septic patients to those with septic shock suggests that oxidative stress may be implicated in the cellular damage observed during sepsis. The Nox family can be responsible for inflammation, injury, and possibly tissue repair occurring in septic shock [18]. In an LPS-induced septic model, Nox4-knockdown mice had decreased production of inflammatory mediators compared to mice overexpressing Nox4, data supporting the crucial role of Nox4 as a key component of the inflammatory response to sepsis [11]. In patients with pneumonia, Nox2 activation is implicated in oxidative stress and subsequent myocardial damage [19].

The upregulation of Nox2 and the related oxidative stress in patients with sepsis and septic shock admitted to ED can have different clinical implications. First, the levels of sNox2-dp and serum TxB2 and the percentage of HBA may be considered suitable markers of the disease. A trend in higher serum sNox2-dp and serum thromboxane B2 between septic patients who survived and those who died has been observed. It did not achieve the statistical significance, probably due to the low sample size. Their prognostic role should be confirmed in further larger studies. These parameters are low-cost and easy to be implemented in the emergency room and may provide both diagnostic and prognostic information in septic patients. Finally, the identification of specific pathways may guide future targeted and individualized therapy in patients with sepsis and high redox state activation.

No significant differences in echocardiographic parameters between patients with sepsis and with septic shock were found, except for IVC collapsibility, maybe due to a small sample size effect, or for the differences in hemodynamic optimization including volume administration in patients with septic shock.

Even if echocardiography has been proposed as the fifth pillar in bedside physical examination, its value in terms of mortality reduction in sepsis and septic shock has not been definitely proven [20].

Nevertheless, bedside echocardiography is extremely useful in septic patients to further assess the hemodynamic state and fluid responsiveness, as different forms of cardiac dysfunction may be present: LV systolic and/or diastolic dysfunction, right ventricular dysfunction, and hyperdynamic LV function with or without LVOT outflow obstruction [21].

This study has some limitations. First, the sample size is low, but it is the largest study evaluating the redox status and platelet function in patients with sepsis/septic shock. Moreover, some drugs such as aspirin and nonsteroidal anti-inflammatory drugs (NSAIDs) may affect platelet activation; however, no differences in previous aspirin, NSAID, and statin use have been detected between patients with sepsis and those with septic shock. We cannot exclude the idea that other factors may have impacted on the oxidative stress status of some included patients; however, data about all concomitant medications have been collected and did not differ between the groups of patients. Finally, not all patients with sepsis or septic shock consecutively admitted to ED of our hospital were included in the study; the collection of a blood sample for the detection of oxidative stress parameters was not possible before fluid replacement therapy and inotrope administration for all patients with sepsis/septic shock admitted to the ED of our hospital.

However, our study has also some strengths. All patients underwent blood sample collection for the dosages of parameters of the redox status and platelet aggregation before volume expansion and inotrope administration to avoid potential interferences with the results. Moreover, they were prospectively observed until 30 days from the ED admission. Finally, we were able to compare the redox status and platelet aggregation of septic patients to healthy volunteers, highlighting the differences in these parameters in the healthy status and in the presence of sepsis.

In conclusion, the oxidative stress status and platelet function in patients with sepsis and septic shock might be easy-to-use disease markers in the ED and may have a prognostic value, allowing the early identification of patients at high risk at 30-day mortality. Regulating Nox-mediated signaling and platelet function may effectively modulate inflammatory responses: further studies are needed to investigate the potential role of Nox signaling as a therapeutic target.

## Figures and Tables

**Table 1:** Clinical characteristics of all patients and comparison between patients with sepsis and those with septic shock.

Variables	Healthy controls (*N* = 10)	Overall (*N* = 33)	Patients with sepsis (*N* = 20)	Patients with septic shock (*N* = 13)	*p*
Demographics					
Male	5 (50%)	16 (48.5%)	9 (45%)	7 (53.8%)	0.619
Age, median (IQR)	68.5 (62-73)	75 (54-85)	74 (54-82.5)	79 (52.5-86.5)	0.888
Previous hospitalization, last 3 months	—	14 (42.4%)	7 (35%)	7 (53.8%)	0.284
Previous antibiotic therapy, last 30 days	—	7 (21.1%)	6 (30%)	1 (7.7%)	0.126
Comorbidities					
Cardiovascular disease	—	19 (57.6%)	11 (55%)	8 (61.5%)	0.710
Chronic heart failure	—	9 (27.3%)	4 (20%)	5 (38.5%)	0.245
Diabetes	—	7 (21.1%)	5 (25%)	2 (15.4%)	0.509
Chronic kidney disease	—	15 (45.5%)	7 (30%)	8 (61.5%)	0.073
Hepatic disease	—	4 (12.1%)	1 (5%)	3 (23.1%)	0.120
Neurologic disease	—	8 (24.2%)	5 (25%)	3 (23.1%)	0.9
COPD	—	9 (27.3%)	6 (20%)	3 (23.1%)	0.833
Solid cancer	—	7 (21.1%)	5 (25%)	2 (15.4%)	0.509
Splenectomy	—	1 (3%)	0	1 (7.7%)	0.208
Aspirin use	—	10 (30.3%)	7 (35%)	3 (23.1%)	0.466
Charlson Comorbidity Index	—		4.5 (1-7)	8 (2-9)	0.298
Source of infection					
Respiratory tract	—	9 (27.3%)	6 (30%)	3 (23.1%)	0.663
Genitourinary tract	—	8 (24.2%)	4 (20%)	4 (30.8%)	0.481
Abdomen	—	8 (24.2%)	4 (20%)	4 (30.8%)	0.481
Skin and soft tissue	—	1 (3%)	1 (5%)	0	0.413
Meningitis	—	1 (3%)	1 (5%)	0	0.413
Bone	—	1 (3%)	1 (5%)	0	0.413
Intravascular device	—	1 (3%)	0	1 (7.7%)	0.208
Others^∗^	—	4 (12.1%)	3 (15%)	1 (7.7%)	0.530
PICC	—	3 (9.1%)	2 (10%)	1 (7.7%)	0.822
CVC	—	2 (6.1%)	1 (5%)	1 (7.7%)	0.751
NIV	—	4 (12.1%)	2 (10%)	2 (15.4%)	0.643
Severity scores of sepsis					
SAPS-II	—	34 (23.5-42)	32 (17.5-38)	37 (31-51)	0.024
qSOFA	—	2 (1-2)	1 (0-2)	2 (2-2.5)	0.002
SOFA score	—	6 (4-10)	4.5 (2-6)	10 (7.5-12)	<0.001
Use of inotropes	—	9 (27.3%)	—	9 (69.2%)	<0.001
Mortality	—	9 (27.3%)	3 (15%)	6 (46.2%)	0.050

COPD = chronic obstructive pulmonary disease; CVC = central venous catheter; IQR: interquartile range; NIV = noninvasive ventilation; PICC = peripheral inserted central catheter. ^∗^Others include 1 malaria (sepsis), 1 mononucleosis (sepsis), and 2 bacteremia with an unknown source of infection (1 sepsis and 1 septic shock).

**Table 2:** Comparison of parameters of the redox status and platelet function among healthy controls, septic patients who survived, and those who died during 30 days after the septic episode.

Variables	Septic patients who survived (*N* = 24)	Septic patients who died (*N* = 9)	*p*
Platelet aggregation (%)	66.5 (58.5-71.5)	73 (60-80)	0.166
Serum thromboxane B2 (pmol/L)	129.5 (120-193.75)	190 (132.5-220)	0.094
Serum sNox2-dp (pg/mL)	20.5 (18-28)	29 (19-32.5)	0.309
Serum H_2_O_2_ scavenger (HBA) (%)	45 (29.25-53.5)	35 (24.5-52.5)	0.619

Nox: NADPH oxidase; HBA: hydrogen peroxide breakdown activity; sNox2-p: soluble NOX2-derived peptides.

## Data Availability

The data used to support the findings of this study are available from the corresponding author upon request.

## Supplementary Materials

Supplementary Materials Supplementary Table S1: source of infection and etiology of the 33 episodes of sepsis. Supplementary Table S2: echocardiographic parameters in patients with sepsis and with septic shock. Supplementary Table S3: echocardiographic parameters in septic patients who survived and those who died.
