# Supplementary material for: Interplay between Nox2 Activity and Platelet Activation in Patients with Sepsis and Septic Shock: A Prospective Study
Source: Oxid Med Cell Longev. 2020 Oct 27;2020:4165358. doi: 10.1155/2020/4165358 (PMC7641261; doi:10.1155/2020/4165358)
Supplement: Supplementary Materials — Supplementary Table S1: source of infection and etiology of the 33 episodes of sepsis. Supplementary Table S2: echocardiographic parameters in patients with sepsis and with septic shock. Supplementary Table S3: echocardiographic parameters in septic patients who survived and those who died. [file 4165358.f1.docx]

**TABLE S1.** Source of infection and etiology of the 33 episodes of sepsis.

| Source of infection | N of cases | Etiological diagnosis |
| --- | --- | --- |
| Respiratory tract infections | N=9 | *S. aureus* N=1  *Enterobacter cloaceae+ A. baumannii* N=1  *Klebsiella pnuemoniae* N=2 |
| Urinary tract infections | N=8 | *E. coli* N=3  *Klebsiella pnuemoniae* N=2  *Proteus* spp N=1  *Enterococcus faecium* N=1 |
| Intra-abdominal infections | N=8 | *E. coli + Proteus* spp N=1  *E. coli* N=2  *Enterococcus faecium* N=1  *Klebsiella* spp + *E. coli* N=1 |
| Skin and soft tissue infection | N=1 | - |
| Osteomyelitis | N=1 | *S. aureus* N=1 |
| CVC-related infection | N=1 | *Candida krusei* N=1 |
| Meningitis | N=1 | *Neisseria meningitidis* N=1 |
| Viral infection | N=1 | Mononucleosis syndrome from EBV |
| Malaria | N=1 | *Plasmodium malariae* |
| Others (BSI of unknown source) | N=2 | *Atopobium parvulum*  *S. aureus* |

**TABLE S2.** Echocardiographic parameters in patients with sepsis and with septic shock.

|  | **Patients with sepsis**  **N=17** | **Patients with septic shock**  **N=10** | **p value** |
| --- | --- | --- | --- |
| Heart rate, bpm | 89 (71.0; 117.0) | 105.5 (77.5; 125.2) | 0.467 |
| End-diastolic diameter, mm | 48.7 (45.2; 56.0) | 47.0 (38.5; 53.3) | 0.380 |
| End-systolic diameter, mm | 40.0 (30.5; 42.5) | 28.0 (20; 40) | 0.245 |
| Interventricular septum, mm | 9.7 (8.0; 10.8) | 9.0 (8.3; 10.5) | 0.653 |
| Posterior wall, mm | 8.0 (7.8; 9.0) | 8.4 (7.5; 9.0) | 0.625 |
| LV mass, g | 160.1 (110.1; 200.8) | 128.8 (106.6; 176.9) | 0.651 |
| Relative wall thickness, ratio | 0.32 (0.31; 0.37) | 0.35 (0.29; 0.43) | 0.571 |
| End-diastolic volume, ml | 111.5 (86.4; 149.3) | 100.0 (64.3; 134.3) | 0.384 |
| End-systolic volume, ml | 50.3 (39.7; 74.0) | 45.0 (32.3; 70.4) | 0.336 |
| Ejection fraction, % | 51.0 (42.0; 57.7) | 46.0 (37.2; 55.0) | 0.571 |
| LVOT, mm | 1.98 (1.76; 2.07) | 1.91 (1.65; 2.15) | 0.799 |
| LVOT VTI, cm | 26.3 (19.7; 29.1) | 17.6 (16.3; 26.3) | 0.056 |
| Stroke volume, ml | 64.9 (59.9; 90.5) | 60.7 (44.4; 74.8) | 0.120 |
| Cardiac output (L/min) | 6.4 (5.5; 8.2) | 6.2 (4.3; 7.0) | 0.198 |
| PW mitral E wave, cm/s | 87.8 (54.9; 94.8) | 91.8 (56.5; 108.6) | 0.655 |
| PW mitral A wave, cm/s | 80.5 (64.4; 108.0) | 58.7 (36.4; 74.7) | 0.116 |
| E/A ratio | 0.71 (0.66; 1.41) | 1.51 (0.54; 2.29) | 0.896 |
| Tissue Doppler E', cm/s | 10.8 (7.8; 14.3) | 16 (12.5; 17.5) | 0.142 |
| E/E' ratio | 6.2 (5.5; 7.9) | 7.0 (6.0; 7.8) | 0.713 |
| Right ventricular end-diastolic diameter, mm | 33.1 (30.7; 35.0) | 34.0 (31.3; 37.9) | 0.430 |
| Tricuspid annular plane systolic excursion, mm | 19.7 (18.3; 23.8) | 19.0 (17.0; 21.7) | 0.329 |
| Pulmonary artery systolic pressure, mmHg | 31.8 (24.0; 37.0) | 30.0 (25.0; 35.5) | 1.0 |
| IVC maximum diameter on inspiration, mm | 15.0 (1.27; 1.91) | 15.0 (1.27; 1.93) | 0.930 |
| IVC minimum diameter on expiration, mm | 0.77 (0.50; 1.14) | 0.97 (0.58; 1.82) | 0.265 |
| IVC collapsibility index, % | 49.2 (27.8; 62.5) | 23.6 (11.5; 46.4) | ***0.035*** |

***Legend***. IVC = inferior vena cava; LV = left ventricular; LVOT = left ventricular outflow tract; PW = pulsed wave Doppler; VTI = velocity time integral. Data are median (1st; 3rd quartile).

**TABLE S3.** Echocardiographic parameters in septic patients who survived and those who died.

|  | **Septic patients who survived**  **N=20** | **Septic patients who died**  **N=7** | **p value** |
| --- | --- | --- | --- |
| Heart rate, bpm | 96.5 (80.5; 125.5) | 77 (69.0; 99.0) | 0.109 |
| End-diastolic diameter, mm | 48.4 (44.6; 55.7) | 51.0 (45.0; 54) | 0.716 |
| End-systolic diameter, mm | 38.0 (35.0; 41.0) | nd | -- |
| Interventricular septum, mm | 9.6 (8.0; 10.0) | 10.8 (9.2; 11.0) | 0.097 |
| Posterior wall, mm | 8.0 (7.8; 8.7) | 8.6 (8.0; 9.0) | 0.269 |
| LV mass, g | 135.5 (98.7; 172.1) | 187.4 (126.4; 206.3) | 0.130 |
| Relative wall thickness, ratio | 0.33 (0.29; 0.38) | 0.32 (0.31; 0.35) | 1.0 |
| End-diastolic volume, ml | 103.7 (85.0; 141.3) | 123.0 (77.1; 138.7) | 0.717 |
| End-systolic volume, ml | 46.9 (39.0; 69.9) | 71.0 (30.7; 93.2) | 0.413 |
| Ejection fraction, % | 50.0 (43.5; 54.5) | 42.0 (33.5; 56.5) | 0.563 |
| LVOT, mm | 1.91 (1.74; 2.06) | 2.0 (1.85; 2.15) | 0.332 |
| LVOT VTI, cm | 21.5 (17.1; 28.0) | 21.0 (20.0; 32.1) | 0.223 |
| Stroke volume, ml | 60.9 (43.5; 70.3) | 63.1 (47.6; 74.8) | 0.462 |
| Cardiac output (L/min) | 6.1 (4.0; 6.7) | 5.6 (2.9; 8.0) | 0.950 |
| PW mitral E wave, cm/s | 64.7 (50.0; 91.2) | 91.8 (83.2; 118.4) | 0.055 |
| PW mitral A wave, cm/s | 71.5 (58.7; 84.0) | 120.0 (75.0; 142.5) | 0.368 |
| E/A ratio | 0.71 (0.66; 1.51) | 0.88 (0.75; 1.97) | 0.791 |
| Tissue Doppler E', cm/s | 12.4 (9.2; 16.7) | 12.5 (9.9; 14.6) | 0.794 |
| E/E' ratio | **5.8 (5.6; 6.8)** | **7.8 (7.2; 13.2)** | **0.037** |
| Right ventricular end-diastolic diameter, mm | **33.0 (30.8; 35.0)** | **38.0 (36.5; 41.0)** | **0.033** |
| Tricuspid annular plane systolic excursion, mm | 19.0 (18.1; 23.6) | 19.0 (17.5; 19.7) | 0.465 |
| Pulmonary artery systolic pressure, mmHg | 30.0 (24.0; 37.0) | nd | -- |
| IVC end-inspiratory diameter, mm | 15.0 (13.0; 19.6) | 13.5 (12.0; 15.0) | 0.160 |
| IVC end-expiratory diameter, mm | 9.8 (5.0; 15.3) | 6.5 (5.7; 8.0) | 0.333 |
| IVC collapsibility index, % | 32.0 (15.4; 58.3) | 48.7 (42.8; 53.8) | 0.463 |

***Legend***. IVC = inferior vena cava; LV = left ventricular; LVOT = left ventricular outflow tract; PW = pulsed wave Doppler; VTI = velocity time integral. Data are median (1st; 3rd quartile).
